# Why tyrosine kinase inhibitor resistance is common in advanced gastrointestinal stromal tumors

**DOI:** 10.12688/f1000research.2-152.v1

**Published:** 2013-07-09

**Authors:** Cristian Tomasetti, George D Demetri, Giovanni Parmigiani

**Affiliations:** 1 Department of Biostatistics, Harvard School of Public Health, Boston MA, 02215, USA; 2 Department of Biostatistics and Computational Biology, Dana-Farber Cancer Institute, Boston MA, 02215, USA; 3 Ludwig Center for Cancer Research at Dana-Farber Cancer Institute and Harvard Medical School, Boston MA, 02215, USA; 4 Center for Sarcoma and Bone Oncology, Dana-Farber Cancer Institute, Boston MA, 02215, USA; 5 Current affiliation: Division of Biostatistics & Bioinformatics, Sidney Kimmel Comprehensive Cancer Center, Johns Hopkins University School of Medicine, Baltimore, MD , 21205-2013 , USA; 6 Current affiliation: Department of Biostatistics, Johns Hopkins Bloomberg School of Public Health, Baltimore, MD, 21205-2179 , USA

## Abstract

**Background:** Most patients with advanced gastrointestinal stromal tumors (GIST) develop drug resistance to tyrosine kinase inhibitors (TKIs) within two years of starting therapy, whereas most chronic myeloid leukemia (CML) patients in chronic phase still exhibit disease control after a decade on therapy. This article aims to explain this divergence in long term outcomes.

**Methods and results:** By combining clinical and experimental observations with mathematical formulas we estimate that, in advanced GIST, the genetic changes responsible for resistance are generally already present at disease detection.

**Conclusion:** This result has relevant clinical implications by providing support for the exploration of combination therapies.

## Introduction

Gastrointestinal stromal tumors (GIST) are sarcomas arising in the muscle wall of the gastrointestinal tract. The majority of GISTs are driven by activating mutations in the receptor tyrosine kinases KIT or PDGFR-α, whose aberrant signaling induces uncontrolled proliferation and decreased apoptosis ^1^. Because of the small number of driving genetic mutations, GISTs represent a paradigm for kinase-driven solid tumors, and offer one of the best models to shed light on fundamental questions about cancer, providing a critical understanding of more genomically complex solid tumors.

Tyrosine kinase inhibitors (TKIs), such as imatinib and sunitinib, block the aberrant activation of KIT, leading to major clinical benefits of objective response and durable disease control. Imatinib represents the standard first-line therapy for this disease when it is surgically incurable. However, imatinib does not cure advanced GIST ^2^. Moreover, while the large majority of patients treated with imatinib do not show signs of primary resistance (disease progression within the first six months of therapy), secondary resistance to imatinib emerges in at least half the patients after two years of therapy and in more than 80% of patients after seven years ^1, 3, 4^.

Imatinib was originally introduced to treat chronic myeloid leukemia (CML), because of its specificity for the TK domain in the *bcr-abl* translocation gene, which characterizes the disease. Imatinib represents the standard first-line therapy and has led to dramatic improvements in outcomes for CML patients in the chronic phase: at six years the estimated event-free survival is 83%, and an estimated 93% of treated patients are free from progression ^5^. Importantly, secondary resistance is far less frequent in CML ^5^.

In this article we consider two fundamental, clinically relevant, but still unanswered questions: Why do patients with advanced GIST often relapse and does secondary resistance originate before or during TKI treatment?

## Materials and methods

In order to obtain estimates for the probability of at least one drug resistant GIST cell being present at the time the tumor reaches a given diameter, and before the introduction of the TKI, we used formula (5) in Tomasetti *et al.* ^6^:

$$P_{R}=1-e^{-uM\left(\frac{1-a/2-b}{1-a-b}\right)\frac{l\left(1-a-b\right)}{d+lb}\ln\left(\frac{l\left(1-a-b\right)}{l\left(1-a-2b\right)-d}\right)}.$$

This formula estimates the probability of having resistant mutants **P _R_** in a tumor of size **M**, (number of cells), and is derived by counting the number of divisions required for the tumor to reach that size. It is assumed that, at each cell division, there is a small probability **u** that one of the daughter cells is hit by a mutation known to induce drug resistance. The parameter **l** and **d** are the birth and death rates for the cell population, while **a** and **b** are the probabilities for the cells’ mode’s of division. This mathematical result is based on standard assumptions and has been successfully used to predict the development of acquired resistance to targeted epidermal growth factor receptor (EGFR) blockade in colorectal cancer ^7^. In order to apply this formula we used parameter estimates available in the literature, as follows.

The somatic point mutation rate **u** can be estimated to be between 10 ^-9^ and 10 ^-8^ per base per cell division ^8, 9^. There are many known point mutations causing resistance to imatinib ^10^. To obtain a conservative bound, we assumed a value of 10 point mutations in our calculations, though the actual number could be higher ^11^. Overall, the probability of a point mutation causing drug resistance in GIST should then be at least 10 ^-8^ per cell division. It has been estimated that 10 ^-9^ cancer cells are present per cm ^3^ of tumoral mass ^12^. As our goal was to generate an unfavorable scenario to our hypothesis, and since stromal tissue and other types of cells may be present, we halved this amount. Long-term drug resistance requires secondary mutations to be present in cells that are long-lived or able to self-renew ^13^. KIT is known to have anti-apoptotic activity, thus contributing to cell lifespan ^14^. Alternatively, resistance could arise from mutations in rare cells possessing stem cell attributes. Interstitial cells of Cajal progenitors are a potential candidate ^15^. Their frequency is estimated to be 6.2 × 10 ^-3^ of all cells ^16^. To be conservative, we only considered the stem cell compartment and set all other parameters of the main formula (5) in Tomasetti *et al*. ^6^ to zero.

**Figure 1.**
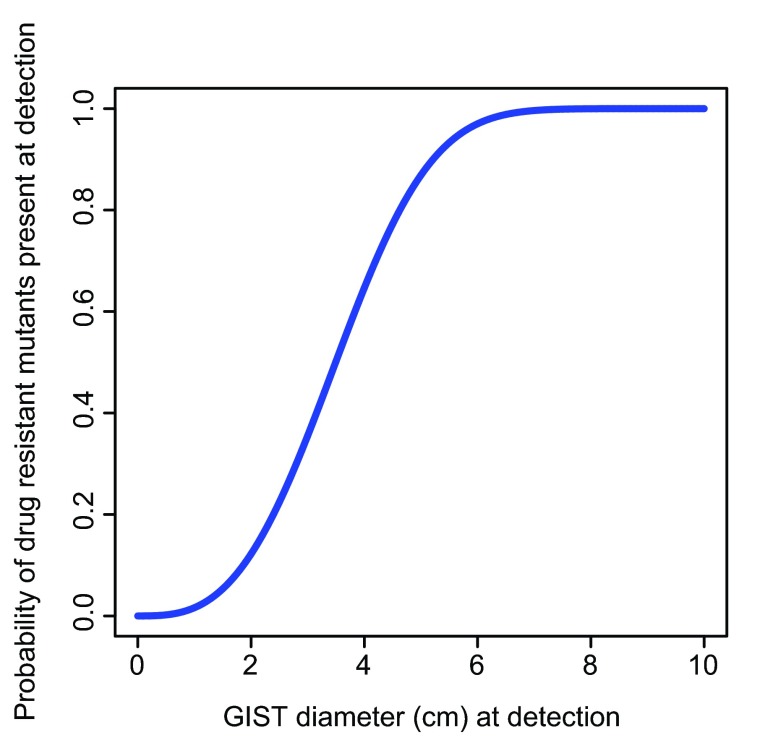
The estimated lower bound for the probability that mutant cells resistant to imatinib are already present in a gastrointestinal stromal tumor (GIST) at time of treatment, as a function of tumor diameter.

Using these results and parameters values, we can derive the lower bound of Figure 1 for the probability of at least one drug resistant GIST cell being present at the time the tumor reaches a given diameter, and before the introduction of the TKI. Because we selected parameters to generate an unfavorable scenario to our hypothesis, we expected the actual curve to be above the curve of Figure 1. The calculation and the figure were obtained using the freely available R software (version 2.15.3) ^17^.

## Results

We combined experimental data with our mathematical formulas ^6^ that recently have been successfully used to predict the development of acquired resistance to targeted EGFR blockade in colorectal cancer ^7^. By so doing, we have obtained the relationship shown in Figure 1 between the tumor diameter at detection, and the probability that the tumor already harbors a resistant mutant at that time (see Materials and methods for the derivation of Figure 1). For example, for GISTs with diameters of 2 and 6 cm we estimate this probability to be equal to 0.12 and 0.97, respectively.

We now bring these elements to bear in interpreting recent clinical observations. The size of a GIST at presentation may vary between 1 and >40 cm in diameter ^1^. For example, in a clinical trial (NCT00237185) of 147 patients with unresectable or metastatic GIST expressing KIT and treated with imatinib, 75% of the patients had a GIST whose diameter was larger than 7 cm at treatment ^18^. Based on Figure 1, we would expect the large majority of these patients to have resistant mutant cells already present by the time of GIST detection and therefore to develop resistance during therapy. In fact, only about 20% of the patients in this clinical trial were still progression-free after five and a half years from the start of imatinib treatment ^18^.

The combination of experimental and clinical data with our mathematical estimates implies then that secondary resistance in patients with advanced GIST is to be expected and is due to the large size of the tumor at detection time. That is, mathematical estimates indicate that mutations responsible for secondary resistance are already present before the start of the treatment in patients with large tumor sizes, a key factor in explaining the high relapse of advanced GIST patients to TKI.

## Discussion

It has been recently suggested that secondary resistance may be the result of the treatment itself rather than pretreatment mutations ^4^. It is worth considering this issue further. Secondary mutations are not usually found at detection using conventional Sanger sequencing techniques ^4^. However, these techniques are not sensitive for rare events. Because imatinib has not yet selected for resistant mutants, they may remain extremely rare, and thus are likely missed by the assays used for mutation analysis. Thus, the fact that mutants are rarely found at detection does not contradict the possibility that the point mutations did occur before treatment. Using our mathematical formulas again ^4^, we estimate that if resistance is present in a GIST of 2 cm in diameter, only approximately 1 out of 10 ^8^ tumor cells will be drug resistant. Most importantly, while resistance may also originate during treatment, this does not exclude the fact that random point mutations can occur during the pre-treatment phase at each cell division. We have shown that the estimated number of resistant mutants produced before the start of the treatment is sufficient to explain the observed clinical data. This logic also holds for the development of tertiary resistant clones that emerge within, on average, six months of starting second-line therapy with sunitinib following failure of imatinib ^19^. Thus, our calculations, while not addressing the problem of primary resistance, are able to explain secondary resistance in advanced GIST.

Our prediction that patients with smaller GISTs at detection will have a smaller probability of progression has been already supported by the clinical correlation of tumor size at presentation with improved outcomes on imatinib ^18^. With sufficient data, it may also be possible to observe a relation between tumor diameter at detection and probability of resistance that is similar in shape to Figure 1, since a resistant clone originating before therapy should eventually grow to a detectable size. Lastly, in the future, with next-generation DNA sequencing techniques, it may become possible to detect the presence of secondary mutations that represent a very small fraction of cells at initial presentation.

Our results, if confirmed, would have important clinical implications. A therapy using a combination of TKIs (for example by adding a new agent with novel spectrum activity to imatinib) would select only for those cells which have been hit by two point mutations, each causing resistance to one of the drugs. We estimate that such a probability is very small for realistic values of the GIST diameter (not considering cross resistance). Therefore, combination therapy could drastically reduce the development of drug resistance caused by point mutations. Also, earlier detection of GIST offers the potential to provide measurable improvements in outcome, as long as it can result in a sufficiently large reduction in the average diameter at detection.
