# Supplementary material for: Why tyrosine kinase inhibitor resistance is common in advanced gastrointestinal stromal tumors
Source: F1000Res. 2013 Jul 9;2:152. [Version 1] doi: 10.12688/f1000research.2-152.v1 (PMC3892920; doi:10.12688/f1000research.2-152.v1)
Supplement: Peer review history

# Peer review: Referee response for version 1

**Published:** 2013-07-18

**Authors:** Gerald Prager

**Affiliations:** 1 Department of Medicine, University of Vienna, Vienna, Austria

Tomasetti *et al*. were combining clinical and experimental observations with mathematical formulas to estimate that in advanced GIST, the genetic changes responsible for resistance are already present from the beginning of treatment. The model is of highest interest, especially in respect of second and third line treatment with targeted therapies. However, prospective validation is required before this model can be incorporated in combination drug trials.

I have read this submission. I believe that I have an appropriate level of expertise to confirm that it is of an acceptable scientific standard.

# Peer review: Referee response for version 1

**Published:** 2013-07-18

**Authors:** Shiro Urayama

**Affiliations:** 1 Department of Internal Medicine, University of California Davis Medical Center, CA, USA

This is a brief report depicting a mathematical model based estimation of presence of chemoresistant cells in gastrointestinal stromal tumor (GIST). The model incorporates the notion of the presence of rare cells possessing stem cell attributes (cancer stem cells) among heterogeneous cells within such mass, contributing to the chemotherapeutic resistance. On the basis of genetic point mutation as a source for the resistance, the probability formula for existence of resistant cells at a tumor size was constructed. Despite some constraints, the article presents reasonable assumptions providing for the lower bound of the probability. Based on this model, many of the clinical index GIST mass has significant likelihood of containing a tyrosine kinase inhibitor resistant cell at the time of the discovery. If future clinical findings support the assumptions made, the model would demonstrate a rational for combination therapeutics.

**Specific points:**

Title and Abstract: Appropriate

Article content: Methods and approaches are described appropriately and references made to the main formula utilized

Conclusions: Appropriate

Data: Appropriate as included

**Minor item:** 

Materials and Methods second paragraph, 5th sentence should have *“109 cancer cells”* instead of *“10-9 cancer cells are present per cm3 of tumor mass”.*

I have read this submission. I believe that I have an appropriate level of expertise to confirm that it is of an acceptable scientific standard.

# Response

**Published:** 2013-11-11

**Authors:** Cristian Tomasetti

**Affiliations:** Johns Hopkins University, USA

We thank the referee. It should read " *estimated that 10 ^9^ cells*".
